# Structural Optimization of Platinum Drugs to Improve the Drug-Loading and Antitumor Efficacy of PLGA Nanoparticles

**DOI:** 10.3390/pharmaceutics14112333

**Published:** 2022-10-29

**Authors:** Maria B. Sokol, Margarita V. Chirkina, Nikita G. Yabbarov, Mariia R. Mollaeva, Tatyana A. Podrugina, Anna S. Pavlova, Viktor V. Temnov, Rania M. Hathout, Abdelkader A. Metwally, Elena D. Nikolskaya

**Affiliations:** 1 N. M. Emanuel Institute of Biochemical Physics of Russian Academy of Sciences, Moscow 119334, Russia; 2 Chemistry Department, Lomonosov Moscow State University, Moscow 119234, Russia; 3 Department of Pharmaceutics and Industrial Pharmacy, Faculty of Pharmacy, Ain Shams University, Cairo 11566, Egypt; 4 Department of Pharmaceutics, Faculty of Pharmacy, Health Sciences Center, Kuwait University, P.O. Box 24923, Jabriya 13110, Kuwait

**Keywords:** PLGA, nanoparticles, molecular docking, PASS, drug release, carboplatin, antitumor efficacy, drug delivery

## Abstract

Currently, molecular dynamics simulation is being widely applied to predict drug–polymer interaction, and to optimize drug delivery systems. Our study describes a combination of in silico and in vitro approaches aimed at improvement in polymer-based nanoparticle design for cancer treatment. We applied the PASS service to predict the biological activity of novel carboplatin derivatives. Subsequent molecular dynamics simulations revealed the dependence between the drug–polymer binding energy along with encapsulation efficacy, drug release profile, and the derivatives’ chemical structure. We applied ICP-MS analysis, the MTT test, and hemolytic activity assay to evaluate drug loading, antitumor activity, and hemocompatibility of the formulated nanoparticles. The drug encapsulation efficacy varied from 0.2% to 1% and correlated with in silico modelling results. The PLGA nanoparticles revealed higher antitumor activity against A549 human non-small-cell lung carcinoma cells compared to non-encapsulated carboplatin derivatives with IC_50_ values of 1.40–23.20 µM and 7.32–79.30 µM, respectively; the similar cytotoxicity profiles were observed against H69 and MCF-7 cells. The nanoparticles efficiently induced apoptosis in A549 cells. Thus, nanoparticles loaded with novel carboplatin derivatives demonstrated high application potential for anticancer therapy due to their efficacy and high hemocompatibility. Our results demonstrated the combination of in silico and in vitro methods applicability for the optimization of encapsulation and antitumor efficacy in novel drug delivery systems design.

## 1. Introduction

**Figure 1:**
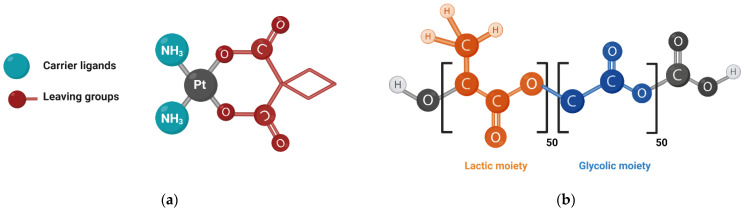
(**a**) Carboplatin structure; (**b**) Structure of PLGA 50/50 with carboxylic terminal group.

Pt-based drugs find an extensive application in the therapy of human neoplasms. Among them, approved worldwide carboplatin reveals significant antitumor efficacy along with low toxicity [1]. However, prescribed regularly for the treatment of testicular, ovarian, head, neck, and small cell lung cancer [2], carboplatin causes serious dose-limiting side effects including myelosuppression and cardiotoxicity [3]. The structure of carboplatin consists of N-donor carrier ligands (NH_3_) and 1,2-cyclobutanedicarboxylate ligands as leaving groups (Figure 1a) [4]. The leaving groups determine the toxicity of the compound—more labile groups display faster hydrolysis, higher reactivity, and toxicity of the whole complex [3,5]. Another concern associated with carboplatin administration is the development of tumor cells’ resistance and poor drug accumulation as a consequence. Probably, this effect occurs due to the dysfunction of proteins, which transport passively Pt-based drugs into cells. However, the complete mechanism is still unclear [6]. Pt-based drugs are non-P-glycoprotein substrates, but prior experiments have showed that cells expressing multidrug resistance-associated protein 2, encoded by the ABCC2 gene, transport the Pt-based drug–glutathione conjugate, thereby reducing intracellular drug concentration [7].

Currently, the strategy of drug delivery with nanoparticles is being applied to overcome drug resistance and improve the safety profile. Drug encapsulation into nanoparticles’ matrix protects the drug from the environment and provides controlled release. In addition, nanoparticles permeate cell membranes through different types of endocytosis including receptor-mediated, which facilitate the overcoming of drug-resistance [8,9,10]. Finally, the enhanced permeability and retention effect, which promotes the penetration of nanoparticles mainly through abnormal tumor vessels, provides selective accumulation of nanoparticles in tumor tissue [11]. Among numerous matrices for drug delivery design, one can be highlighted, poly(lactic-co-glycolic acid) (PLGA, Figure 1b), due to its biodegradability and biocompatibility [12,13]. Moreover, the U.S. Food and Drug Administration approved PLGA-based nanoparticles for parenteral application [14]. The role of nanoformulations in cancer treatment, including PLGA nanoparticles, is extensively reviewed elsewhere [15,16,17,18].

The literature survey revealed several successful formulations of carboplatin-loaded PLGA nanoparticles, but most of them demonstrated low encapsulation efficacy (EE; <50%) demanding high dose application [19,20,21]. Several research groups have reported the enhancement of structure hydrophobicity resulting in increased encapsulation efficiency, making modification of drug properties a useful strategy in delivery systems design [22,23,24]. Since drug physical properties strongly influence EE, it is reasonable to study how structure modification alters EE as well.

**Figure 2:**
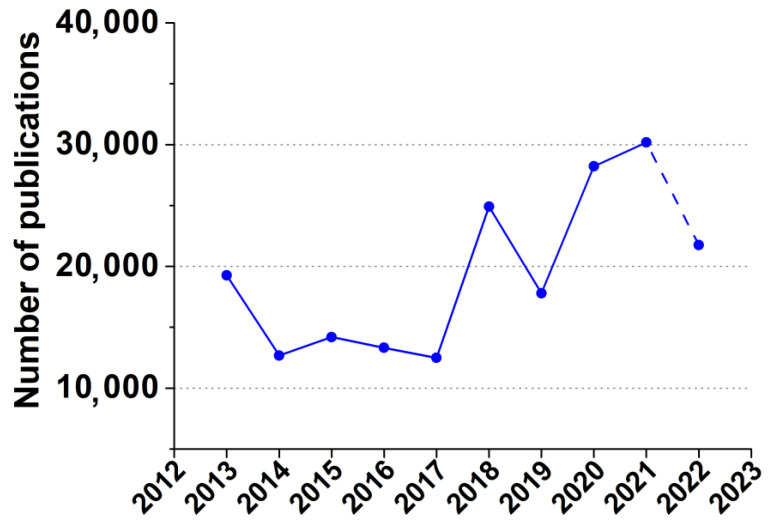
Number of publications on molecular dynamic simulations in drug delivery systems’ design. Data obtained via Dimensions (https://app.dimensions.ai/discover/publication, (accessed on 24 October 2022)) using keywords «molecular dynamic simulations drug delivery systems».

Computer simulations may provide relevant information of system properties. Nowadays, molecular dynamics simulations are used to assess drug interactions with a matrix [25]. This approach is accepted as a modern in silico technique, which provides the binding energy value between drug and matrix based on their physical properties [26]. The application of molecular dynamics simulations in the design of drug delivery systems becomes more popular according to the number of published papers (Figure 2). The group of Hathout revealed interactions of aspirin, prednisolone, resveratrol, and testosterone with a PLGA matrix [27]. Megy and coauthors applied a molecular dynamics study to obtain insight into the molecular mechanisms of the poly (lactic acid) nanoparticle formation and their interactions with vitamin E and Toll-like receptor agonists [25]. Stipa and coauthors studied PLGA-quinone system to support its application against SARS-CoV-2 [28]. Dobhal and coauthors applied the approach to vary PLGA monomers ratio to reveal the best one for coumarin-6 encapsulation [29].

Even if drug moiety remained intact, ligands may alter in vitro and in vivo properties of the designed compound. In this case, screening of biological activity with computational tools, such as PASS (Prediction of Activity Spectra for Substances), is a useful step for improving the development process [30,31].

**Figure 3:**
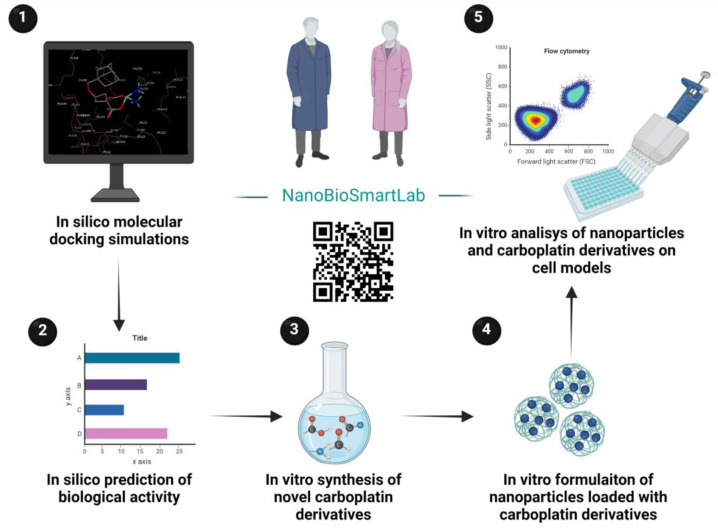
Research graphical workflow.

In the present research, we used molecular dynamics simulations to assess the influence of different carboplatin ligands on affinity to PLGA and EE. Next, we applied an online service PASS to predict the anticancer activity of modified carboplatin structures. Finally, we compared the obtained in silico results with the experimental data (%DL and EE of formulated nanoparticles, drug release, and cytotoxicity against A549, H69, and MCF-5 cells) to conclude whether the combination of in silico and in vitro methods is precise enough and useful for PLGA nanoparticle design and the prediction of properties and biological activity. Figure 3 shows a graphical workflow of our research.

## 2. Materials and Methods

### 2.1. Materials

Poly(d,l-lactide-co-glycolide) (PLGA) with the ratio of lactic and glycolic monomers of 50:50, inherent viscosity 0.2 dL/g in HFIP, MW 17,000–21,000), was purchased from LACTEL Absorbance Polymers (Birmingham, AL, USA). D-mannitol, Polyvinyl alcohol (PVA, MW 30,000–70,000), carboplatin, cisplatin (cis-[PtCl_2_(NH_3_)_2_]) were purchased from Sigma-Aldrich (St. Louis, MO, USA). 1-Adamantaneacetic acid, Lauric acid, Valeric acid were purchased from Sigma-Aldrich (St. Louis, MO, USA). Chloroform, methylene chloride, and acetone were purchased from Ruskhim (Moscow, Russia). Dimethyl sulfoxide (DMSO) and phosphate-buffered saline (PBS) were purchased from Amreso (Solon, OH, USA). Triton X100, Ethylenediaminetetraacetic acid (EDTA) were purchased from Serva (Heidelberg, Germany). HNO_3_ was purchased from KhimMed (Moscow, Russia). Sodium dodecyl sulfate (SDS) was obtained from PanReac AppliChem (Chicago IL, USA). Heparin was obtained from Khimmed Syntes (Moscow, Russia). Trypsin and fetal bovine serum (FBS) were purchased from Hyclone (Logan, UT, USA). DMEM and RPMI-1640 were purchased from Gibco (Waltham, MA, USA). 3-(4,5-dimethyl-thiazol-2yl)-2,5-diphenyltetrazoliumbromide (MTT), was purchased from Sigma-Aldrich (St. Louis, MO, USA). Annexin V-FITC/PI kit was purchased from Biolegend (San Diego, CA, USA). All other chemicals were used as HPLC grade or extra pure grade. Water was purified in a Millipore Milli-Q Plus system (Darmstadt, Germany).

### 2.2. All-Atom Molecular Dynamics Simulations of Polymer Carriers

GROMACS version 4.6.5 software package (University of Groningen, Groningen, Netherlands) was used to perform molecular dynamics simulations for the studied polymer carriers. PLGA 50:50 matrix was constructed from 60 molecules of PLA and 60 molecules of PGA tetramers. The CHARMM36 force field parameters were obtained using the CGenFF server (https://cgenff.paramchem.org/ (accessed on 5 March 2020)). The steepest descent integrator was applied for energy minimization of the systems. Systems were simulated under a full periodic boundary conditions and a timestep of 2 fs. A cutoff length, set to 12 Å, defined both van der Waals and electrostatic interactions. PME was used as the treatment method for electrostatic interactions. LINCS algorithm was used for bond constraining. The simulations were allowed to run until an equilibrium state was reached where a constant system temperature, energy, and density were observed. The total simulation time was 3.2 ns [27,32].

#### 2.2.1. Molecular Docking of the Investigated Molecules on the Virtually Constructed Carriers

The software builder of MOE^®^ version 2014.0901 (Chemical Computing group Inc., Montreal, QC, Canada) was used to generate the 3-D structure of the investigated molecules. MMFF94x forcefield was utilized to minimize the energy of the molecules and to obtain the corresponding mdb file needed for docking [33]. The bond length was adjusted in the constructed structures using Avogadro software which was also used to calculate the Marsilli–Gastiger partial charges.

MOE^®^ version 2014.0901 (Chemical Computing group Inc., Montreal, QC, Canada) docking software was used for this task. First, locating possible target positions in the carrier was performed using the MOE’s site finder tool. After, dummy atoms were added [34]. Each of the investigated molecules was placed at these locations utilizing the triangle matcher placement method. Flexible docking was conducted adopting the ASE scoring function. A mean value of the top five poses binding energies and their SD were calculated [32,35,36].

#### 2.2.2. Calculating the Main Descriptors of the Investigated Drugs

In order to explain the differences in docking scores observed for the studied drugs, some crucial constitutional, electronic, and topological descriptors were calculated. The selected descriptors were the molecular weight, LogP (o/w), total polar surface area, number of H-atom donors and acceptors, and molecular globularity and molecular flexibility [37]. The descriptors were calculated using MOE^®^ version 2014.0901 (Chemical Computing group Inc., Montreal, QC, Canada) utilizing the molecules’ mdb files generated using the same software [38,39].

### 2.3. PASS-Based In Silico Assessment of Compounds’ Activity

Prediction of possible antitumor activity was assisted with PASS online serves [31]. The data on compound structures were inputted as SMILES generated with molecular structure drawing software ACD/ChemSketch.

### 2.4. Synthesis of Carboplatin Derivatives

**Figure 4:**
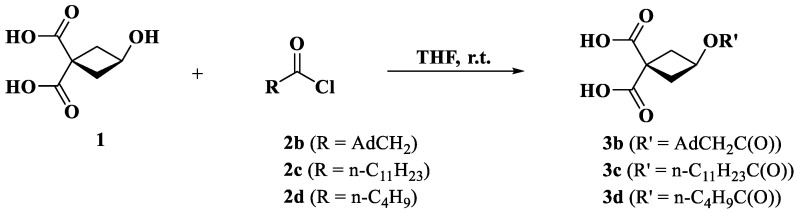
Synthesis of ligands for new platinum complexes.

Figure 4 describes the synthesis of carboplatin derivatives. We used 3-hydroxycyclobutane-1,1-dicarboxylic acid (**1**) as the starting compound to obtain new ligands **3** (**b**–**d**) via acylation with acid chlorides **2** (**b**–**d**) under optimized conditions. Acid **(1)** was prepared according to a previously described method [40].

**Figure 5:**
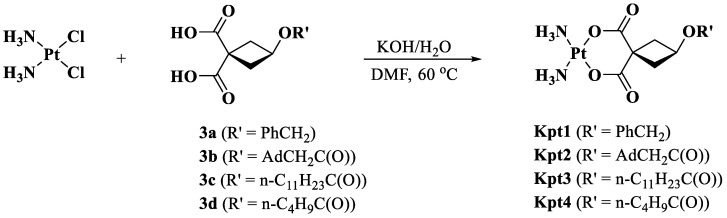
Synthesis of carboplatin derivatives Kpt1-4.

We synthesized novel carboplatin derivatives Kpt1-4 from cisplatin *cis*-[PtCl_2_(NH_3_)_2_] and ligands **3** (**a**–**d**) according to previously reported procedure (Figure 5) [41].

We confirmed the synthesized structures with NMR and high-resolution mass spectra analysis.

All solvents were purified and degassed before use. Acid chlorides **2 (b–d)** were prepared according to standard method [42]. Ligand **3a** is the precursor in the synthesis of 3-hydroxycyclobutane-1,1-dicarboxylic acid **1**.

#### 2.4.1. Synthesis of Ligands **3** (**b**–**d**)

General procedure for the synthesis of ligands **3** (**b**–**d**)

3-hydroxycyclobutane-1,1-dicarboxylic acid **1** (0.2 g, 1.25 mmol) was dissolved in THF (5 mL), and the corresponding acid chloride **2** (**b**–**d**) (6.25 mmol) was added. The reaction mixture was stirred at room temperature until TLC indicated the total consumption of acid **1**. The solvent was evaporated under reduced pressure. The residue was kept at −24 °C for crystallization for 1 h. Cold petroleum ether was added to the residual solid. A precipitated product was filtrated off and dried under vacuum.

3-(2-((3r,5r,7r)-adamantan-1-yl)acetoxy)cyclobutane-1,1-dicarboxylic acid **3b**

Synthesis was performed according to general procedure. 2-((3r,5r,7r)-adamantan-1-yl)acetyl chloride **2b** (1.33 g, 6.25 mmol) were used. Yield: 0.23 g (54%) as a white solid.

^1^H NMR (CD_3_OD, δ): 1.64–1.77 (m, 12H, Ad), 1.94–1.98 (br.m, 3H, Ad), 2.05 (s, 2H, OC(O)CH_2_), 2.53–2.59 (m, 2H, CH_2_ of cyclobutyl), 2.88–2.94 (m, 2H, CH_2_ of cyclobutyl), 4.96 (br.s, 2H, COOH), 5.00 (q, ^3^J_HH_ = 7.3 Hz, 1H, CH of cyclobutyl)).

^13^C NMR (CD_3_OD, δ): 30.22 (Ad), 34.01 (OC(O)CH_2_), 37.94 (Ad), 38.53 (2 CH_2_ of cyclobutyl), 43.62 (Ad), 48.41 (C(COOH)_2_), 49.65 (Ad), 65.24 (CH of cyclobutyl), 172.85, 174.35, 174.72 (C=O).

HRMS-ESI: m/z calcd. for (C_18_H_24_O_6_+H)^+^ 337.1646, found 337.1640.

3-(dodecanoyloxy)cyclobutane-1,1-dicarboxylic acid **3c**

Synthesis was performed according to general procedure. Dodecanoyl chloride **2c** (1.37 g, 6.25 mmol) were used. Yield: 0.393 g (92%) as a white solid.

^1^H NMR (CDCl_3_, δ): 0.88 (t, 3H, ^3^J_HH_ = 6.8 HZ, C_10_H_20_CH_3_), 1.22–1.33 (br.m, 16H, C_8_H_16_CH_3_), 1.57–1.64 (m, 2H, CH_2_); 2.30 (t, 2H, ^3^J_HH_ = 7.6 Hz, OC(O)CH_2_); 2.69–2.74 (m, 2H, CH_2_ of cyclobutyl); 3.03–3.08 (m, 2H, CH_2_ of cyclobutyl); 5.14 (q, ^3^J_HH_ = 7.3 Hz, 1H, CH of cyclobutyl); 11.32 (br.s, 2H, C(COOH)_2_).

^13^C NMR (CDCl_3_, δ): 14.09 (CH_3_), 22.65, 24.76, 29.06, 29.19, 29.30, 29.40, 29.56, 31.88 (CH_2_), 34.10 (OC(O)CH_2_), 37.33 (2 CH_2_ of cyclobutyl), 46.87 (C(COOH)_2_), 63.39 (CH of cyclobutyl), 173.52, 175.91,176.60 (C=O).

HRMS-ESI: m/z calcd. for (C_18_H_30_O_6_+Na)^+^ 365.1935, found 365.1927.

3-(pentanoyloxy)cyclobutane-1,1-dicarboxylic acid **3d**

Synthesis was performed according to general procedure. Pentanoyl chloride **2d** (0.75 g, 6.25 mmol) were used. Yield: 0.216 g (71%) as a white solid.

^1^H NMR (CD_3_OD, δ): 0.92 (t, 3H, ^3^J_HH_ = 7.3 Hz, CH_3_), 1.30–1.39, 1.52–1.61 (m, 2H, CH_2_), 2.31 (t, 2H, ^3^J_HH_ = 7.4 Hz, OC(O)CH_2_), 2.53–2.58 (m, 2H, CH_2_ of cyclobutyl), 2.86–2.92 (m, 2H, CH_2_ of cyclobutyl), 5.01 (q, ^3^J_HH_ = 7.3 Hz, 1H, CH of cyclobutyl), 5.28 (br.s, 2H, OH).

^13^C NMR (CD_3_OD, δ): 14.20 (CH_3_), 23.30, 28.11 (CH_2_), 34.70 (OC(O)CH_2_), 38.39 (2 CH_2_ of cyclobutyl), 48.23 (C(COOH)_2_), 65.30 (CH of cyclobutyl), 174.27, 174.67, 174.89 (C=O).

HRMS-ESI: m/z calcd. for (C_11_H_16_O_6_+Na)^+^ 267.0839, found 267.0850.

#### 2.4.2. Synthesis of Complexes **Kpt1**–**4**

General procedure for the synthesis of the cis-diammineplatinum (II) complexes Kpt1-4

Cisplatin *cis*-[PtCl_2_(NH_3_)_2_] (0.050 g, 0.167 mmol) was dissolved in DMF (3 mL) at 90 °C. Corresponding l,l-cyclobutanedicarboxylic acid **3** (**a**–**d**) (0.167 mmol) and a freshly prepared solution of KOH (0.019 g, 0.335 mmol) in water (3 mL) was added to the solution of cisplatin cooled to 60 °C. The reaction mixture stirred for 24 h at 60 °C in an open flask.

After cooling to room temperature, the reaction mixture was treated with acetone (20 mL) and cooled down to 5 °C for 1 h. A precipitated product was filtrated off, washed with distilled water, acetone, and dried under vacuum.

Complex **Kpt1.** Synthesis was performed according to general procedure. 3-(benzyloxy)cyclobutane-1,1-dicarboxylic acid **3a** (0.042 g, 0.167 mmol) were used. Yield: 0.041 g (52%) as a white solid.

^1^H NMR (DMSO, δ): 2.34–2.45 (m, 2H, CH_2_ of cyclobutyl); 3.09–3.14 (m, 2H, CH_2_ of cyclobutyl), 3.79 (q, ^3^J_HH_ = 7.21 Hz, 1H, CH of cyclobutyl), 4.18 (br.s, 6H, 2NH_3_), 4.36 (s, 2H, OCH_2_Ph), 7.26–7.37 (m, 5H, Ar).

^13^C NMR (DMSO, δ): 38.88 (2 CH_2_ of cyclobutyl), 48.41 (C(COO)_2_), 67.07 (CH of cyclobutyl), 68.87 (OCH_2_Ph), 127.41, 127.61, 128.24, 138.49 (Ph), 176.94, 177.28 (COO^−^).

HRMS-ESI: m/z calcd. for (C_13_H_18_N_2_O_5_Pt+Na)^+^ 499.0735, found 499.0720.

Complex **Kpt2.** Synthesis was performed according to general procedure. 3-(2-((3r,5r,7r)-adamantan-1-yl)acetoxy)cyclobutane-1,1-dicarboxylic acid **3b** (0.056 g, 0.167 mmol) were used. Yield: 0.054 g (57%) as a white solid.

^1^H NMR (DMSO, δ): 1.53–1.68 (m, 12H, Ad), 1.91–1.95 (br.m, 3H, Ad), 2.01 (s, 2H, OC(O)CH_2_), 2.51–2.55 (m, 2H, CH_2_ of cyclobutyl), 3.22–3.27 (m, 2H, CH_2_ of cyclobutyl), 4.21 (br.s, 6H, 2NH_3_), 4.66 (q, ^3^J_HH_ = 7.3 Hz, 1H, CH of cyclobutyl).

^13^C NMR (DMSO, δ): 27.93 (Ad), 32.29 (OC(O)CH_2_), 36.23 (Ad), 38.55 (2 CH_2_ of cyclobutyl), 41.76 (Ad), 47.97 (Ad), 49.38 (C(COO)_2_), 63.40 (CH of cyclobutyl), 170.38, 176.39, 176.75 (C=O).

HRMS-ESI: m/z calcd. for (C_18_H_28_N_2_O_6_Pt+Na)^+^ 585.1466, found 585.1445.

Complex **Kpt3.** Synthesis was performed according to general procedure. 3-(dodecanoyloxy)cyclobutane-1,1-dicarboxylic acid **3c** (0.057 g, 0.167 mmol) were used. Yield: 0.049 g (52%) as a white solid.

^1^H NMR (DMSO, δ): 0.85 (t, 3H, ^3^J_HH_ = 6.7 Hz, CH_3_), 1.20–1.27 (br. m, 16H, C_8_H_16_), 1.45–1.52 (m, 2H, CH_2_); 2.25 (t, 2H, ^3^J_HH_ = 7.3 Hz, OC(O)CH_2_); 2.51–2.55 (m, 2H, CH_2_ of cyclobutyl); 3.21–3.26 (m, 2H, CH_2_ of cyclobutyl); 4.18 (br.s, 6H, 2NH_3_), 4.67 (q, ^3^J_HH_ =7.4 Hz, 1H, CH of cyclobutyl);

^13^C NMR (DMSO, δ): 13.99 (CH_3_), 22.12, 24.43, 28.45, 28.73, 28.90, 29.01, 29.02, 31.31 (CH_2_), 33.42 (OC(O)CH_2_), 38.43 (2 CH_2_ of cyclobutyl), 49.34 (C(COOH)_2_), 63.69 (CH of cyclobutyl), 172.54, 176.45, 176.78 (C=O).

HRMS-ESI: m/z calcd. for (C_18_H_34_N_2_O_6_Pt+H)^+^ 569.2116, found 569.2111.

Complex **Kpt4**. Synthesis was performed according to general procedure. 3-(pentanoyloxy)cyclobutane-1,1-dicarboxylic acid **3d** (0.041 g, 0.167 mmol) were used. Yield: 0.048 g (61%) as a white solid.

^1^H NMR (DMSO, δ): 0.86 (t, 3H, ^3^J_HH_ = 7.3 Hz, CH_3_), 1.23–1.32, 1.45–1.52 (m, 2H, CH_2_), 2.26 (t, 2H, ^3^J_HH_ = 7.3 Hz, OC(O)CH_2_), 2.51–2.56 (m, 2H, CH_2_ of cyclobutyl), 3.21–3.26 (m, 2H, CH_2_ of cyclobutyl), 4.21 (br.s, 6H, 2NH_3_), 4.67 (q, ^3^J_HH_ = 7.3 Hz, 1H, CH of cyclobutyl).

^13^C NMR (DMSO, δ): 13.65 (CH_3_), 21.61, 26.57 (CH_2_), 33.17 (OC(O)CH_2_), 38.45 (CH_2_ of cyclobutyl), 49.37 (C(COO)_2_), 63.71 (CH of cyclobutyl), 172.57, 176.47, 176.80 (C=O).

HRMS: m/z calcd. for (C_11_H_20_N_2_O_6_Pt+H)^+^ 471.1021; found 471.1012.

### 2.5. NMR Spectra Analysis

^1^H and ^13^C NMR spectra were recorded on Bruker Avance 400 (400 and 100 MHz, respectively), Agilent 400-MR spectrometers (400 and 100 MHz, respectively). Residual signals of solvents were used as a reference (^1^H: CDCl_3_, δ 7.26, DMSO-d6, δ 2.49, CD_3_OD, δ 3.31. ^13^C: CDCl_3_, δ 77.1, DMSO-d6, δ 39.52, CD_3_OD, δ 49.0).

### 2.6. High Resolution Mass Spectra Analysis

High resolution mass spectra (HRMS) were recorded on an Agilent LC/MSD 1100 SL instrument with atmospheric pressure electrospray ionization (AP-ESI) in the positive ion detection mode (ion trap mass analyzer). Registration conditions: the nebulizer gas temperature (nitrogen) 300 °C at a rate of 12 L/min, the source potential 5000 V, the capillary outlet potential 150 V.

### 2.7. Nanoparticles Formulation

NPs were formulated via the single emulsion–solvent evaporation method [43,44]. Briefly, 10 mg of Kpt (Kpt1, Kpt2, Kpt3 or Kpt4) were dissolved in 0.5 mL of DMFA and 100 mg of PLGA were dissolved in 2.5 mL of chloroform. Then, solution of Kpt was added to PLGA solution and stirred 10 min. The mixture was added dropwise to 1% PVA solution (15 mL). The emulsion was homogenized (Ultra-Turrax^®^ T-25 basic, Staufen im Breisgau, Germany) followed by evaporation during 50 min to remove the organic solvent (IKA HB10, Staufen im Breisgau, Germany). Further, NPs were centrifuged for 30 min at 15.000× *g*, 4 °C (BECKMAN J2-2, Palo Alto, CA, USA). The resulting pellet was resuspended in 10 mL of distilled water, and filtered via glass filter with a porosity of 47–111 μm. Finally, 10% (*w*/*w*) of D-mannitol was added to the suspension followed by lyophilization. Obtained NPs were stored at 4 °C until further use.

Blank nanoparticles were formulated as described above except for Kpt addition.

### 2.8. Measurement of Size, Zeta Potential, and Polydispersity Index (PDI) Measurement

Dynamic light scattering (DLS) was used to analyze size and PDI of NPs. Electrophoretic light scattering was used to measure zeta potential of NPs. All the parameters were analyzed with Nano-ZS ZEN 3600 (Malvern-Instruments, Worcestershire, UK) [45].

Prior to analysis, samples were diluted with distilled water at a concentration of 1 mg/mL. For each sample, three independent measurements were made [46].

### 2.9. Nanoparticles’ Morphology

Visualization of Kpt-NPs was performed with transmission electron microscopy (TEM; Tecnai Osiris, FEI, Hillsboro, OR, USA). Prior to analysis samples were diluted with distilled water at a concentration of 1 mg/mL. A drop of sample was placed on a 3 mm copper grid covered with formvar film and dried for 30 min at 25 °C.

### 2.10. Drug Loading and Encapsulation Efficiency Measurement

Drug loading (DL) and encapsulation efficiency (EE) of Kpt-loaded NPs were analyzed by ICP-MS (Agilent 7500C, Agilent Technologies, Tokyo, Japan). The sample introduction system consisted of a robust Babington nebulizer with a Scott spray chamber (Agilent Technologies, Tokyo, Japan) cooled by a Peltier element (2 °C). The ChemStation software package (Agilent Technologies, version G1834B; Santa Clara, CA, USA) was used to acquire and process the data. Samples were diluted with the mixture of 0.1% Triton X-100–1% HNO_3_. The same solution was used as the mobile phase of the mass spectrometer.

The DL of Kpt-NP was determined by dissolving approximately 5 mg of NPs in 1 mL of ICP-MS diluent.

Kpt EE was analyzed according to the following protocol: 5 mg of NPs were resuspended in 2 mL of distilled water and centrifuged at 5000× *g* during 5 min at room temperature (5417R, Eppendorf, Germany). The supernatant was removed and the procedure was repeated with the pellet. The precipitate was freeze-dried and analyzed by ICP-MS.

DL (%) and EE (%) were calculated according to Equations (1) and (2):

DL = (weight of remained drug in the particles/weight of particles) × 100(1)

EE = (100 − ((total amount of drug − actual amount of drug in precipitate)/total amount of drug)) × 100(2)

### 2.11. Animals

Female Chinchilla rabbits were obtained from Center for Laboratory Animal Breeding Krolinfo Ltd., Russia. The animals were kept in the vivarium with a natural light cycle and access to food and water ad libitum. The study was organized and implemented in accordance with the requirements of the Ministry of Health of Russia No. 199 on 1 April 2016 on Approval of the Rules for Good Laboratory Practice and EU Directive 2010/63/EU for animal experiments.

### 2.12. Hemolytic Activity Study

The hemolytic activity study was performed according to Dobrovolskaia and colleagues’ protocol with modifications [47]. Briefly, 4 mL of rabbit blood were collected from marginal ear vein and centrifuged for 5 min at 358× *g* and 4 °C. The pellet containing blood cells was washed with PBS (0.01 M, pH 7.4) three times and suspended in 13 mL of PBS. A total of 200 µL of blank NPs, Kpt4, Kpt5, or Kpt6 at concentration of 10 mg/mL, 0.1 mg/mL, or 0.01 mg/mL was mixed with 200 µL of red blood cell (RBC) suspension in a 1.5 mL tube. The mixture was incubated at 37 °C, under continuous agitation for 3 h and then centrifuged for 5 min at 2240× *g* at room temperature. A total of 150 µL of the supernatants was transferred in a 96-well plate, mixed with 17 µL of 0.6% SDS and incubated for 5 min to convert blood hemoglobin into a low-spin oxidized form—hemihrome. The amount of hemoglobin released from erythrocytes was determined calorimetrically by measuring the absorbance at 540 nm. Hemolysis level was determined according to Equation (3):

Hemolysis (%) = ((absorbance of sample-absorbance of negative control)/(absorbance of positive control-absorbance of negative control)) × 100%(3)

The RBCs were exposed to a 10% Triton X100 or 0.01 M PBS to formulate corresponding positive or negative control. The samples were analyzed in triplicate.

### 2.13. In Vitro Release Study

The in vitro Kpt and Kpt1-Kpt4 release from the NPs was carried out at 37 °C in 0.01M PBS (pH 7.4) supplemented with 0.1% (w/v) Tween 80. A total of 84 plastic tubes (4 NPs batches in triplicate; Safe-Lock tubes 2.0 mL, Eppendorf, Hamburg, Germany) were filled with 5 mg of lyophilized NPs and diluted with 2 mL of 0.01 M PBS. The tubes were placed into a horizontal shaker (37 °C, 100 rpm, MS 3 basic; IKA, Germany). At predetermined time points, samples were withdrawn, centrifuged at 5000× *g* for 5 min at room temperature (5417R, Eppendorf, Germany) and the resulted pellet was freeze-dried. The samples were analyzed in triplicate. The amount of residual Kpt in the NPs was determined by ICP-MS using previously described protocol.

The mathematical modeling of Kpt release profiles from NPs was performed with the DDSolver^®^ add-in program for Microsoft Excel [48].

### 2.14. Cell Culture

The cancer cells line A549 (human non-small-cell lung carcinoma), H69 (human small-cell lung carcinoma), and MSF-7 (human breast adenocarcinoma) were purchased from the American Type Culture Collection (ATCC, Manassas, VA, USA). A549 and MSF-7 cells were maintained in DMEM medium supplemented with 10% fetal bovine serum and gentamycin (50 μg/mL). H69 cells were maintained in RPMI-1640 medium with the same FBS and gentamycin concentrations. Cells were maintained in plastic 25 cm^2^ cell culture flasks at 37 °C in a humidified atmosphere containing 5% CO_2_. A549 cells were plated before reaching 80% confluence using trypsin/EDTA solution.

### 2.15. Cytotoxicity Assay

A549, MCF-7, and H69 cells were plated in 96-well plates (3500, 5000, and 10,000 cells per well, correspondingly) one day before the experiment and incubated under standard conditions. Stock solutions of the samples were dissolved in DMEM (in RPMI-1640 in H69 cells case). Then, samples were dissolved in DMFA, following dilution by media to minimize DMFA toxic effects (>5% *v*/*v*). Kpt substances, nanoparticles, and blank nanoparticles were analyzed in 0.125–200 μM concentration range in triplicates (equivalent to free Kpt concentration) applying standard a MTT test [49]. The plates with H69 cells were additionally centrifuged at 200× *g* for 5 min to sediment formazan crystals prior to medium aspiration. Samples were incubated for 72 h. Cell viability was determined as the percent of untreated control. Mean survival values were calculated in Excel (Microsoft Corporation, Redmond, Washington, DC, USA)) and data visualization was performed in OriginPro (version 2020b, OriginLab Corp., Northampton, MA, USA).

### 2.16. Apoptosis Assay

Necrotic and apoptotic populations were analyzed using annexin V-FITC/propidium iodide (PI) staining. Briefly, 1 × 10^5^ A549 cells were seeded in petri dishes and incubated overnight. After 1 h incubation with the Kpt, Kpt1-Kpt4, and Kpt-NPs, cells were rinsed, harvested, and incubated with 10 μL (5 μg/mL) annexin V-FITC and 10 μL PI (20 μg/mL). After 15 min of incubation, cells were rinsed and immediately analyzed by flow cytometer CyAn ADP (Beckman Coulter, Brea, CA, USA) (λex 488 nm, 613/20 nm bandpass filter for PI and 530/40 for annexin V-FITC).

### 2.17. Statistical Analysis

The results were expressed as mean ± standard deviation (SD). All statistical analyses were performed using GraphPad Prism^®^ v.5.0 (GraphPad software, San Diego, CA, USA) and at a level of significance *p* < 0.05.

## 3. Results and Discussion

### 3.1. In Silico Molecular Docking on PLGA Matrix

In the current research, we considered the second-generation Pt-based drug carboplatin as a model compound since it has been approved world-wide [50] and the comprehensive information on its in vitro and in vivo evaluation is available in the literature [51,52,53].

**Figure 6:**
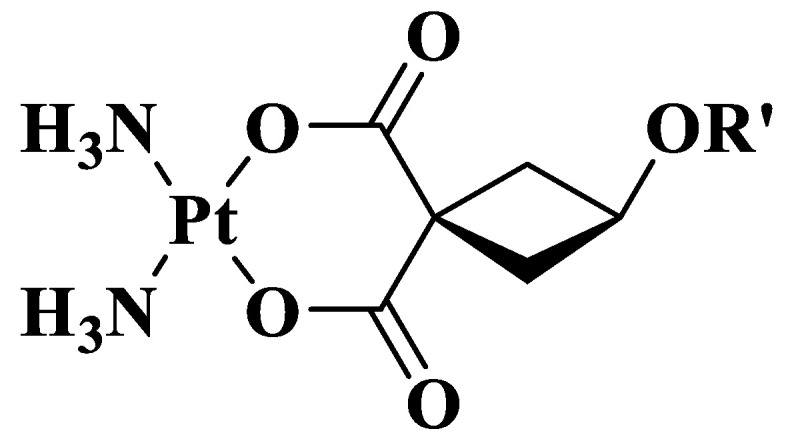
Structure of carboplatin moiety.

We selected several types of ligands with different structural features to add in carboplatin moiety (Figure 6, Table 1): two linear alkyl ligands (Kpt4 and Kpt3), benzyl (Kpt1), and adamantyl (Kpt2) ligands. Selected structures are widely used to modify biologically active compounds [54,55,56,57] and have similar parameters in order to assess the influence of minimum molecular weight and lipophilicity difference on binding affinity to polymer matrix.

We simulated docking of novel carboplatin derivatives on PLGA to determine whether the novel derivatives change its affinity to polymer matrix, compared to intact carboplatin. PLGA with lactic and glycolic ratios 50:50 was considered as a model matrix, since it has the most favorable properties compared to other ratios in drug encapsulation and release kinetics [58].

Table 2 lists the main physico-chemical properties of molecules influenced on drug binding efficacy to polymer. Compared to carboplatin (Kpt), all the derivatives have a higher hydrogen-bond acceptor level, which is due to extra oxygen atoms [59].

In all cases, modification of carboplatin also resulted in increased molecular weight, logP, and molecular flexibility, with Kpt3 molecular flexibility approximately 3.5 times higher, compared to carboplatin. The higher the values of described parameters, the more readily molecules interact with hydrophobic (lactic) moieties and permeate through the highly entangling and viscous polymer matrix.

**Figure 7:**
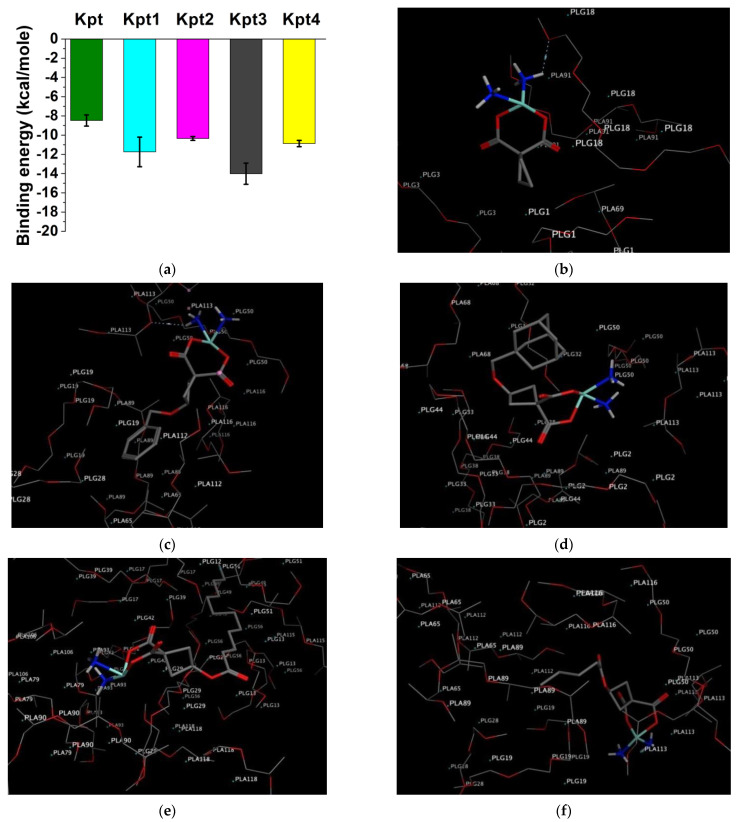
(**a**) Values of binding energy after drug docking on PLGA 50:50; 3D representation of the interaction of (**b**) Kpt, (**c**) Kpt1, (**d**) Kpt2, (**e**) Kpt3, and (**f**) Kpt4 with PLGA.

Figure 7b–f demonstrates the successful docking of carboplatin and its derivatives on PLGA. The docking of the investigated molecules scored the lowest binding energy value (better interactions with the polymer matrix) in the case of Kpt3 (Figure 7a). This is in line with calculated descriptors: maximum values of log *p*, molecular weight, and flexibility along with the minimum globularity. The reversible dependence is due to the thermodynamic features of the involved system [60,61].

Literature data confirmed the influence of calculated molecular descriptors on drug interaction with polymeric matrix. Thus, Forrey and coauthors reported molecular flexibility is a crucial parameter in the strength of binding affinity [62]. Johnstone and Lippard showed that increasing the chain length increases the overall lipophilicity of the complex, facilitating its partition into the PLGA nanoparticle core [23].

In addition, the binding energy of all derivatives was lower than that one of carboplatin, indicating an improved affinity of novel derivatives to the polymer.

### 3.2. PASS-Based In Silico Assessment of Compounds’ Activity

We assessed the anticancer activities of proposed chemical structures using the PASS-online service [63]. The predicted activities are presented as probabilities “to be active” Pa and “to be inactive” Pi calculated for each activity. Pa > Pi indicates possible activity for a compound [64].

**Figure 8:**
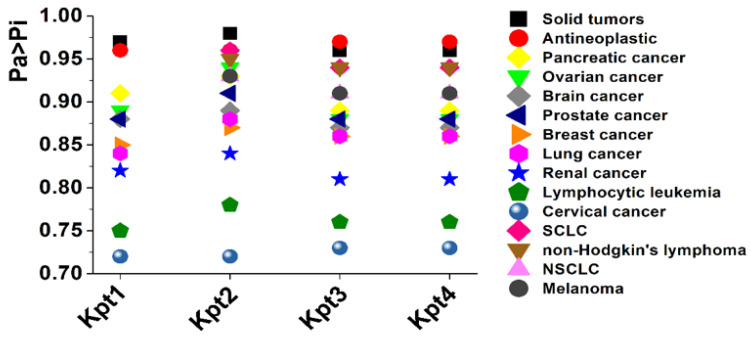
PASS prediction results of anticancer activity. SCLC—small cell lung cancer, NSCLC—non-small cell lung cancer.

Proposed compounds exhibited a wide range of the anticancer activities with Pa > Pi values from 0.73 to 0.98 (Figure 8; Supplementary Tables S1–S4). According to the predictions, all the compounds remained carboplatin activity profile intact: ovarian, breast, and lung cancer [65]. However, Kpt1 lacked SCLC and NSCLC activity. PASS also revealed extra activities related to solid tumors and lymphomas for all of the derivatives (Figure 8; Supplementary Tables S1–S4). In general, the modification of carboplatin moiety with applied moieties remained the anticancer activity of the derivatives.

Next, we synthesized simulated carboplatin derivatives and formulated PLGA nanoparticles loaded with the derivatives to compare the experimental data with the theoretically calculated ones (Figure 4 and Figure 5).

### 3.3. Formulation of Nanoparticles

**Figure 9:**
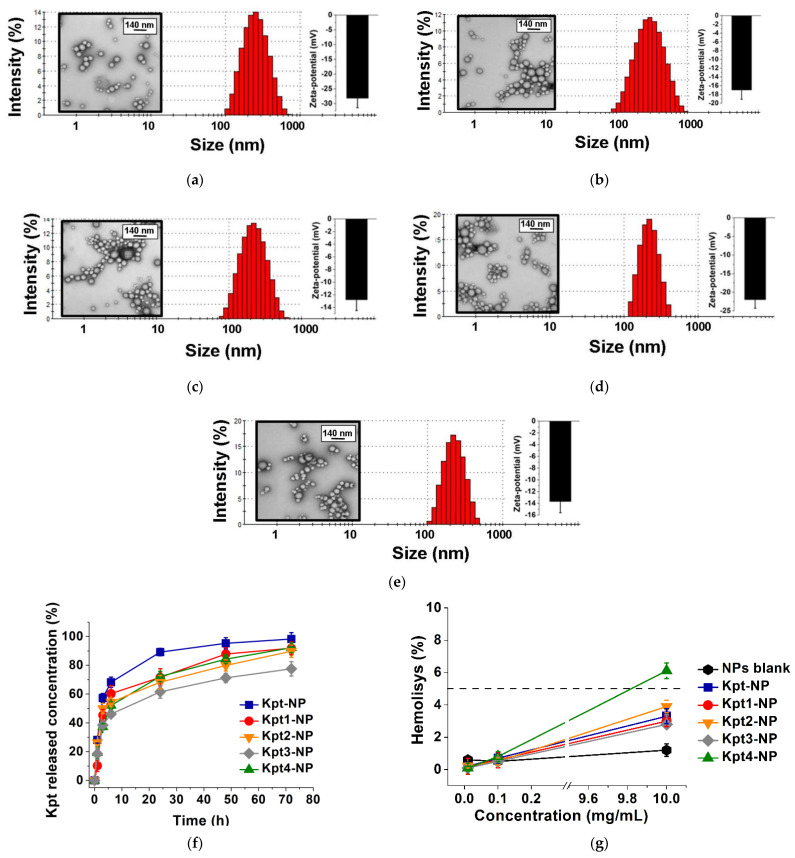
Size distribution, ζ-potential and TEM results of NPs: (**a**) Kpt-NP, (**b**) Kpt1-NP, (**c**) Kpt2-NP, (**d**) Kpt3-NP, (**e**) Kpt4-NP; (**f**) in vitro release profiles of Kpt and Kpt1-4 from NPs in 0.01M PBS pH 7.4. Each point shows mean ± SD (*n* = 3); (**g**) hemolytic activity of designed NPs (mean ± SD, *n* = 3).

We formulated PLGA nanoparticles, loaded with carboplatin and the derivatives via the single emulsion solvent evaporation technique. Designed NPs had a spherical shape and smooth surface, according to TEM results (Figure 9a–e). The size of NPs was in the range of 180–300 nm (Table 3, Figure 9a–e), which is favorable for passive targeting and accumulation in tumors via abnormalities in tumor blood vessels [11]. All the analyzed samples had ζ-potential values from −12.8 mV to −28.0 mV, indicating sufficient colloid stability [66].

We observed a significant increment (*p* < 0.05) of Kpt3-NP entrapment efficacy compared to other formulations, which agreed with binding energy scoring results (Table 3). Kpt1-NP, Kpt2-NP, and Kpt4-NP exhibited negligible DL and EE growth compared to Kpt-NP.

The drug loading trends provide similar pattern: Kpt1-NP, Kpt2-NP, and Kpt4-NP values increased slightly with a sharp increase in Kpt3-NP drug loading. Probably, it is the combination of high flexibility, logP, and molecular weight, which provide a significant increase in drug concentration in PLGA nanoparticles. Thus, we concluded that there was a correlation between the experimental results and simulation trends, making reasonable application of in silico simulation in design of PLGA nanoparticles.

### 3.4. In Vitro Kpt Release

The Kpt and Kpt1-4 release profile demonstrated a biphasic pattern: initial burst release during the first 5 h, followed by a prolonged sustained phase up to 87% in 80 h (Figure 9f), which was often observed in NPs smaller than 200 nm compared to microparticles [67,68]. The initial burst release occurs due to Kpt and Kpt1-4 detachment from NPs surface or release of Kpt and Kpt1-4 molecules placed near the inner surface, easily accessible by hydration [69]. The second release phase showed slow diffusion from PLGA pores or cracks formed due to hydration and degradation process [70].

We performed modeling to determine the influence of drug derivatization on its release kinetics mechanism from PLGA matrix. We used five conventional release mathematical models, each having specific properties. The zero-order model explains an ideal pattern where the drug release rate is dependent only on time and independent on drug concentration [71,72]. The first-order equation expresses the dependence of drug release rate on its concentration [73]. The Higuchi model describes the drug release as insoluble in the release media and non-swelling matrix [74]. The Hickson–Crowell model considers cases where the matrix tends to biodegrade, such as in a hydrolytic or enzymatic way [75]. The Korsmeyer–Peppas equation describes the main transport phenomena caused either by diffusion or swelling [76].

As a criterion of fitting to a release curve, we used the coefficient of determination R^2^. According to results shown in Table 4, the Korsmeyer–Peppas model was best-fitted for describing the release kinetics in NPs, providing R^2^ values > 0.9293.

We evaluated exponent *n* of the Korsmeyer–Peppas model to estimate the correlation between the exponent values and possible release mechanism (Table 5).

The *n* values for all the formulations were below 0.45, which in case of spheres, corresponds to quasi-Fickian diffusion transport [77]. This type of transport indicates the drug release is mainly limited by the drug diffusion, while the polymer swelling influence the drug release insignificantly [77,78].

The increased values of the release rate constant (k) for all formulations compared to Kpt-NP, with Kpt3-NP having the maximum value, is in line with calculated descriptors, mainly molecular flexibility, and molecular globularity (Table 5). The minimum globularity along with high flexibility values result in high diffusion rate through polymer matrix.

### 3.5. Hemolytic Activity Study

Since the synthesized nanoparticles are designed for parenteral administration, it assumes to interact the red blood cells (RBCs) [79]. We studied the hemocompatibility of the formulated nanoparticles via analysis of RBC hemolysis. The concentration range of 0.01–10 mg/mL, selected for analysis, reflects 10-fold higher and lower concentrations of Kpt and Kpt derivative nanoformulations, based on theoretical dose for in vivo application [51]. We interpreted results according ASTM protocol F756-13 [80].

Blank NPs over the concentration range described and all the nanoparticles at the concentrations up to 0.1 mg/mL displayed <2% hemolysis, corresponding to a lack of nonhemolytic effect (Figure 9g). All the nanoformulations at the maximum concentration, except Kpt4-NP, displayed a slight hemolytic effect (from 3 to 4%). Kpt4-NP at the maximum concentration showed slight hemolytic activity (6.1%), but lacked hemolysis at lower concentrations. Our results agreed with previous data: Karanam and coauthors reported carboplatin-loaded nanoparticles based on poly (ε-caprolactone) at 0.02 mg/mL lacked hemolytic activity [81].

Our results proved the safety of intravenous administration of designed nanoparticles at doses assumed for in vivo application.

### 3.6. In Vitro Cytotoxicity Study

We performed the cytotoxic activity study to determine the influence of ligand structure and encapsulation process on compound cytotoxicity. We used non-small cell lung carcinoma cell line A549 as a model system, since one of the main carboplatin indications is lung cancer treatment [82]. We also included in our study two supplementary cells lines—H69 (human small-cell lung carcinoma) and MSF-7 (human breast adenocarcinoma). Thus, the main reasons of these cell lines choice were the high Pa values predicted during PASS analysis for all Kpt derivatives (Figure 8; Supplementary Tables S1–S4) and clinical application of Pt-based drugs in lung and breast cancer treatment.

**Figure 10:**
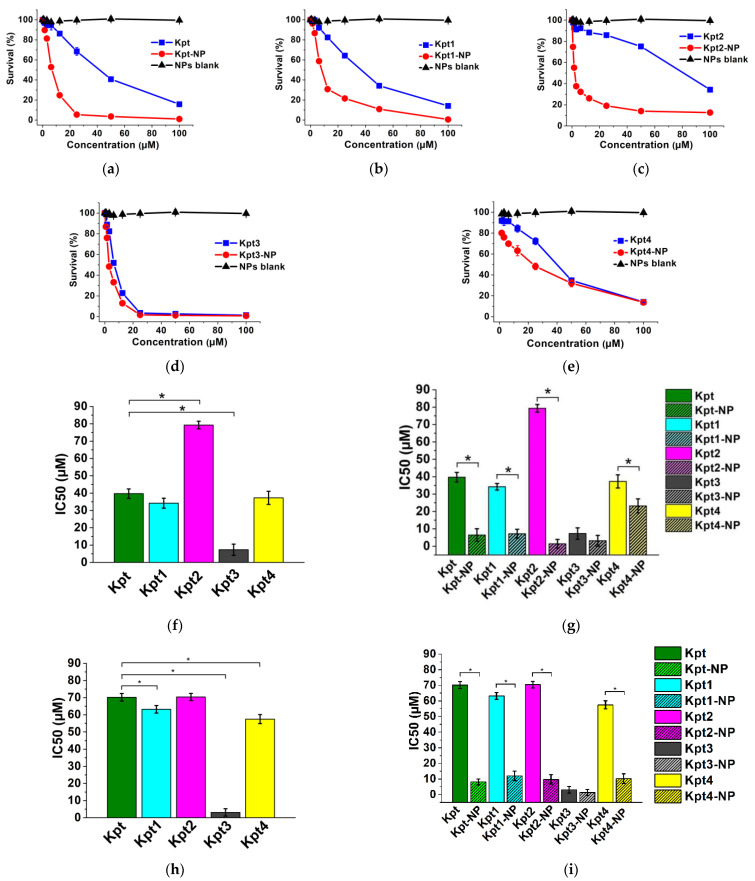

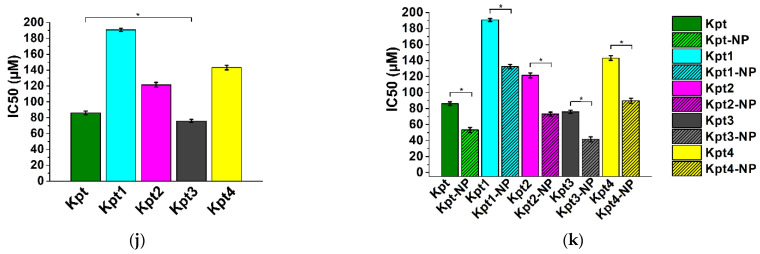
Survival of A549 cells after incubation during 72 h with: (**a**) Kpt and Kpt-NP; (**b**) Kpt1 and Kpt1-NP; (**c**) Kpt2 and Kpt2-NP; (**d**) Kpt3 and Kpt3-NP; (**e**) Kpt4 and Kpt4-NP. IC_50_ values of Kpts and NPs: difference among substances against (**f**) A549 cells, (**h**) H69 cells, (**j**) MCF-7 cells; comparison of Kpts/NPs pairs against (**g**) A549 cells, (**i**) H69 cells, (**k**) MCF-7 cells. Each point shows mean ± SD (*n* = 3); * *p* < 0.05.

Figure 10 shows that a long alkyl chain radical contributed to significant (*p* < 0.05) activity enhancement (IC_50_ for Kpt and Kpt3 were 39.70 µM and 7.32 µM, respectively). Previously, Novohradsky and colleagues reported that a significant anticancer activity increment of Pt-derivatives contained long aliphatic chains [83]. Kpt1 and Kpt4 lacked influence on cytotoxicity compared to carboplatin (IC_50_ for Kpt1, and Kpt4 were 34.20 µM, and 37.30 µM, respectively). The adamantyl ligand (Kpt2) significantly (*p* < 0.05) decreased cytotoxicity (IC_50_ for Kpt2 was 79.30 µM).

Figure 10 and Figures S1 and S2 (Supplementary) show that the substance encapsulation into PLGA nanoparticles generally enhanced cytotoxic activity; but, in the Kpt3 case, where we observed the equivalent with NP activity against all cancer cell lines, MCF-7 cells were outlined, revealing sensitivity specifically to Kpt3-NPs. Enhanced activity of PLGA nanoparticles is probably explained by a different mechanism of cell internalization, which provided efficient cell accumulation of Kpt derivatives [83].

Overall, toxicity values of the compounds were comparable with Kpt and agreed with previously reported data [84,85].

### 3.7. Apoptosis Assay

**Figure 11:**
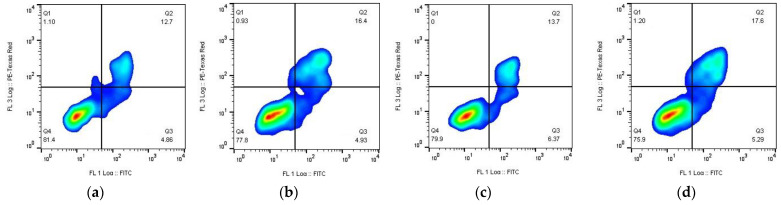

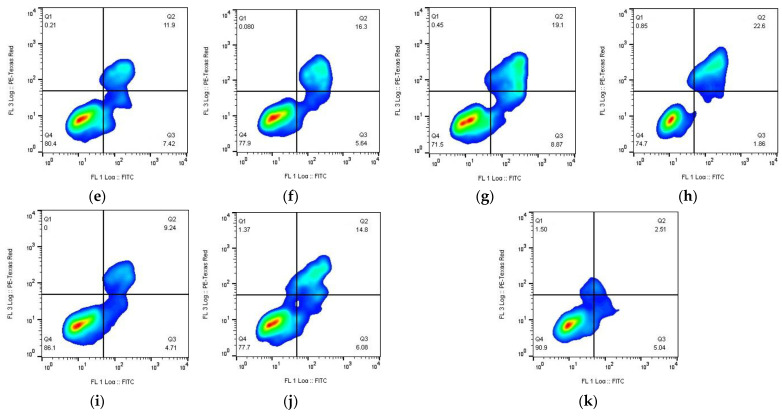
The plots represent apoptotic and necrotic A549 cell populations after 1 h of incubation with the NPs loaded with the derivatives synthesized and the drug substances: (**a**) Kpt and (**b**) Kpt-NP; (**c**) Kpt1 and (**d**) Kpt1-NP; (**e**) Kpt2 and (**f**) Kpt2-NP; (**g**) Kpt3 and (**h**) Kpt3-NP; (**i**) Kpt4 and (**j**) Kpt4-NP; Control represents untreated cells (**k**). The populations characterized with annexin V-FITC fluorescence are early apoptotic and observed at right lower quadrants; the populations characterized with double PI and annexin V-FITC fluorescence are late apoptotic/necrotic and observed at upper right quadrants.

We applied annexin V-FITC/PI double staining to analyze cell death induced by Kpts and Kpt-NPs (Figure 11) [86,87].

We observed Kpt and Kpt-derivative induced damage of A549 cells and formation of late and early apoptotic populations (Figure 11). Up to 28.4% (Q1 + Q2 + Q3 quadrants) (Kpt3) of the cells were at different apoptosis stages after Kpt-derivatives after 1 h of treatment compared to carboplatin—Kpt (18.7% (Q1 + Q2 + Q3)); the late and early apoptotic populations after the NPs application were generally higher than after substance treatment, which could be explained with low Pt-derivative stability in aqueous media and changes in internalization pathways. Among all samples, the Kpt3 (late apoptotic/necrotic cells represented 19.1% of the whole population) and Kpt3-NPs (late apoptotic/necrotic population (Q2) was 22.6%) revealed the most prominent toxicity in contrast with the controls—Kpt substance (Q2 was 12.7%) and Kpt-NPs (Q2 was 16.4%). In contrast, Kpt4 (Q2 was 9.2%) and Kpt4-NPs (Q2 was 14.8%) displayed a lower toxic effect in comparison with Kpt and Kpt-NPs.

Summarizing the apoptosis stimulation analysis, we can build the next arrays displaying the formulations and substances efficacy:

- Substances (early apoptosis (Q3))—Kpt3 (8.9%) > Kpt2 (7.4%) > Kpt1 (6.4%) > Kpt (4.9%) > Kpt4 (4.7%);
- NPs (early apoptosis (Q3))—Kpt4 (6.1%) > Kpt2 (5.6%) > Kpt1 (5.3%) > Kpt (4.9%) > Kpt3 (1.9%);
- Substances (late apoptosis/necrosis (Q2))—Kpt3 (19.1%) > Kpt1 (13.7%) > Kpt (12.7 %) > Kpt2 (11.9%) > Kpt4 (9.24%);
- NPs (late apoptosis/necrosis (Q2))—Kpt3 (22.6%) > Kpt1 (17.6%) > Kpt (16.4%) > Kpt2 (16.3%) > Kpt4 (14.8%).

Overall, Kpt3 and Kpt3-NPs displayed the highest apoptosis stimulation efficacy among all tested samples.

These results displayed high Kpt3 and Kpt1-Kpt3-NPs antitumor efficacy in vitro according to the MTT-test and ability to trigger apoptosis and necrosis in cancer cells, which may be evidence for certain promises for synthesized derivatives and formulated NPs in anticancer application.

## 4. Conclusions

In the current study, we established the relationship between structures of carboplatin derivatives and their binding affinity to PLGA matrix to provide maximum encapsulation efficiency. Molecular dynamics simulations revealed that the ligand with long alkyl chain (C14) had the highest lipophilicity, molecular weight, and flexibility compared to other used ligands, yielding minimum (i.e., favorable) binding energy with PLGA 50:50. Moreover, we observed a certain correlation between calculated binding energy and EE values along with release kinetics. PASS analysis predicted the synthesized derivatives’ anticancer activity profile. Further in vitro experiments confirmed that the encapsulated compounds remained cytotoxic even after loading into nanoparticles. Kpt-derivatives displayed a cytotoxicity level similar with carboplatin, except Kpt3, characterized with higher activity, and Kpt2, characterized with lower activity. Interestingly, NP formulations generally displayed higher activity in both cases—after long (72 h) and short-term (1 h) incubation, which could be explained by two factors—alternative internalizaion pathways and higher derivatives stability inside NPs. Our results indicate that the combined in silico and in vitro approach can be a valuable strategy for the design of PLGA nanoparticles. The described approach can be applied for drug delivery systems design with low drug loading and encapsulation efficacy to increase these parameters.

## 5. Limitations

Besides novel carboplatin derivatives showing good potential to enhance encapsulation efficacy, increased lipophilicity of these compounds will limit its use in further in vivo studies. These water insoluble compounds require the development of a system for parenteral administration to properly compare effects of free substances and nanoformulations in vivo.

## Figures and Tables

**Table 1:** Structures and properties of Kpt ligands.

Compound	Ligand	MW of Ligand	logP of Ligand *
Kpt1	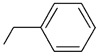 R’ = PhCH_2_	121.16	1.89
Kpt2	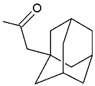 R’ = AdCH_2_C(O)	209.27	3.06
Kpt3	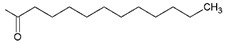 R’ = n-C_11_H_23_C(O)	213.34	5.35
Kpt4	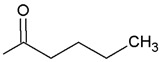 R’ = n-C_4_H_9_C(O)	101.13	1.50

* Calculated with Molinspiration Cheminformatics online service (https://www.molinspiration.com/ (accessed on 5 September 2021)). MW—molecular weight.

**Table 2:** Main physico-chemical descriptors of the investigated molecules.

Compound	Total Polar Surface Area	Number of H-Bond Acceptors	Number of H-Bond Donors	Molecular Globularity	Molecular Flexibility	logP (o/w)	Molecular Weight
Kpt	80.18	2	0	0.17	2.72	−1.80	370.25
Kpt1	32.03	3	0	0.07	4.23	0.74	479.38
Kpt2	48.53	3	0	0.10	4.72	1.52	565.51
Kpt3	65.63	3	0	0.02	9.89	3.38	571.56
Kpt4	63.05	3	0	0.07	5.43	0.29	473.37

**Table 3:** Parameters of Kpt-NPs. Data shown as mean ± SD (*n* = 3).

NPs	Size, nm	PDI	ζ-Potential, mV	DL, %	EE, %
Kpt-NP	215 ± 38	0.243 ± 14	−28.0 ± 3.2	0.16 ± 0.04	54 ± 3
Kpt1-NP	303 ± 26	0.204 ± 13	−17.0 ± 2.1	0.21 ± 0.03	60 ± 2
Kpt2-NP	247 ± 45	0.277 ± 11	−12.8 ± 1.7	0.39 ± 0.03	62 ± 3
Kpt3-NP	180 ± 37	0.127 ± 8	−22.0 ± 2.3	1.07 ± 0.02 *	92 ± 2 *
Kpt4-NP	245 ± 51	0.245 ± 9	−13.7 ± 1.9	0.30 ± 0.03	60 ± 1

*p < 0.05 compared to Kpt-NP, Kpt1-NP, Kpt2-NP, and Kpt4-NP.

**Table 4:** Model fitting of in vitro Kpt and Kpt derivatives release kinetics.

NPs	Zero Order	First Order	Higuchi	Hixson–Crowell	Korsmeyer–Peppas
	R^2^	R^2^	R^2^	R^2^	R^2^
Kpt-NP	0.2652	0.5951	0.6997	0.5271	0.9623
Kpt1-NP	−0.0857	0.4432	0.6534	0.3173	0.9873
Kpt2-NP	0.4219	0.9042	0.8859	0.8291	0.9711
Kpt3-NP	−0.1324	0.6951	0.6267	0.4219	0.9710
Kpt4-NP	0.0052	0.8239	0.6050	0.5170	0.9293

**Table 5:** Calculation results of Korsmeyer–Peppas model parameters.

**Parameters**	**Kpt-NP**	**Kpt1-NP**	**Kpt2-NP**	**Kpt3-NP**	**Kpt4-NP**
n	0.35	0.21	0.32	0.21	0.24
k	19.7	24.0	33.7	35.3	32.7

## Data Availability

Data are contained within the article.

## Supplementary Materials

The following supporting information can be downloaded at: https://www.mdpi.com/article/10.3390/pharmaceutics14112333/s1, Table S1: PASS predicted activity of Kpt1; Table S2: PASS predicted activity of Kpt2; Table S3: PASS predicted activity of Kpt3; Table S4: PASS predicted activity of Kpt4; Figure S1: Survival of H69 cells after incubation during 72 h with: (a) Kpt and Kpt-NP; (b) Kpt1 and Kpt1-NP; (c) Kpt2 and Kpt2-NP; (d) Kpt3 and Kpt3-NP; (e) Kpt4 and Kpt4-NP; Figure S2: Survival of MCF-7 cells after incubation during 72 h with: (a) Kpt and Kpt-NP; (b) Kpt1 and Kpt1-NP; (c) Kpt2 and Kpt2-NP; (d) Kpt3 and Kpt3-NP; (e) Kpt4 and Kpt4-NP.
