# Supplementary material for: Structural Optimization of Platinum Drugs to Improve the Drug-Loading and Antitumor Efficacy of PLGA Nanoparticles
Source: Pharmaceutics. 2022 Oct 29;14(11):2333. doi: 10.3390/pharmaceutics14112333 (PMC9698263; doi:10.3390/pharmaceutics14112333)
Supplement: Supplementary file 1 [file pharmaceutics-14-02333-s001.zip › pharmaceutics-1976606-supplementary.pdf]

SUPPLEMENTARY MATERIALS

Structural Optimization of Platinum Drugs to Improve the Drug-Loading and Antitumor Efficacy of PLGA Nanoparticles

Maria B. Sokol [1,*,†], Margarita V. Chirkina [1,†], Nikita G. Yabbarov [1], Mariia R. Mollaeva [1], Tatyana A. Podrugina [2], Anna S. Pavlova [2], Viktor V. Temnov [2], Rania M. Hathout [3], Abdelkader A. Metwally [3,4] and Elena D. Nikolskaya [1,*]

1 N. M. Emanuel Institute of Biochemical Physics of Russian Academy of Sciences, Moscow 119334, Russia
2 Chemistry Department, Lomonosov Moscow State University, Moscow 119234, Russia
3 Department of Pharmaceutics and Industrial Pharmacy, Faculty of Pharmacy, Ain Shams University, Cairo 11566, Egypt
4 Department of Pharmaceutics, Faculty of Pharmacy, Health Sciences Center, Kuwait University, P. O. Box 24923, Jabriya 13110, Kuwait
* Correspondence: mariyabsokol@gmail.com (M.B.S.); elenanikolskaja@gmail.com (E.D.N.)
† These authors contributed equally to this work.

Table S1. PASS predicted activity of Kpt1.

Pa	*Pi*	*Activity*
0,97	0	Antineoplastic (solid tumors)
0,96	0	Antineoplastic
0,91	0	Antineoplastic (pancreatic cancer)
0,89	0	Antineoplastic (ovarian cancer)
0,88	0	Antineoplastic (brain cancer)
0,88	0	Prostate cancer treatment
0,85	0	Antineoplastic (breast cancer)
0,84	0	Antineoplastic (lung cancer)
0,82	0	Antineoplastic (renal cancer)
0,75	0	Antineoplastic (lymphocytic leukemia)
0,72	0	Antineoplastic (cervical cancer)

Table S2. PASS predicted activity of Kpt2.

Pa	*Pi*	*Activity*
0,98	0	Antineoplastic (solid tumors)
0,96	0	Antineoplastic
0,96	0	Antineoplastic (small cell lung cancer)
0,95	0	Antineoplastic (non-Hodgkin's lymphoma)
0,94	0	Antineoplastic (ovarian cancer)
0,93	0	Antineoplastic (non-small cell lung cancer)
0,93	0	Antineoplastic (pancreatic cancer)
0,93	0	Antineoplastic (melanoma)
0,91	0	Prostate cancer treatment
0,89	0	Antineoplastic (brain cancer)
0,88	0	Antineoplastic (lung cancer)
0,87	0	Antineoplastic (breast cancer)
0,84	0	Antineoplastic (renal cancer)
0,78	0	Antineoplastic (lymphocytic leukemia)
0,72	0	Antineoplastic (cervical cancer)

Table S3. PASS predicted activity of Kpt3.

Pa	*Pi*	*Activity*
0,97	0	Antineoplastic
0,96	0	Antineoplastic (solid tumors)
0,94	0	Antineoplastic (non-Hodgkin's lymphoma)
0,94	0	Antineoplastic (small cell lung cancer)
0,91	0	Antineoplastic (melanoma)
0,91	0	Antineoplastic (non-small cell lung cancer)
0,89	0	Antineoplastic (pancreatic cancer)
0,88	0	Prostate cancer treatment
0,88	0	Antineoplastic (ovarian cancer)
0,87	0	Antineoplastic (brain cancer)
0,86	0	Antineoplastic (breast cancer)
0,86	0	Antineoplastic (lung cancer)
0,81	0	Antineoplastic (renal cancer)
0,76	0	Antineoplastic (lymphocytic leukemia)
0,73	0	Antineoplastic (cervical cancer)

Table S4. PASS predicted activity of Kpt4.

Pa	*Pi*	*Activity*
0,97	0	Antineoplastic
0,96	0	Antineoplastic (solid tumors)
0,94	0	Antineoplastic (non-Hodgkin's lymphoma)
0,94	0	Antineoplastic (small cell lung cancer)
0,91	0	Antineoplastic (melanoma)
0,91	0	Antineoplastic (non-small cell lung cancer)
0,89	0	Antineoplastic (pancreatic cancer)
0,88	0	Prostate cancer treatment
0,88	0	Antineoplastic (ovarian cancer)
0,87	0	Antineoplastic (brain cancer)
0,86	0	Antineoplastic (breast cancer)
0,86	0	Antineoplastic (lung cancer)
0,81	0	Antineoplastic (renal cancer)
0,76	0	Antineoplastic (lymphocytic leukemia)
0,73	0	Antineoplastic (cervical cancer)

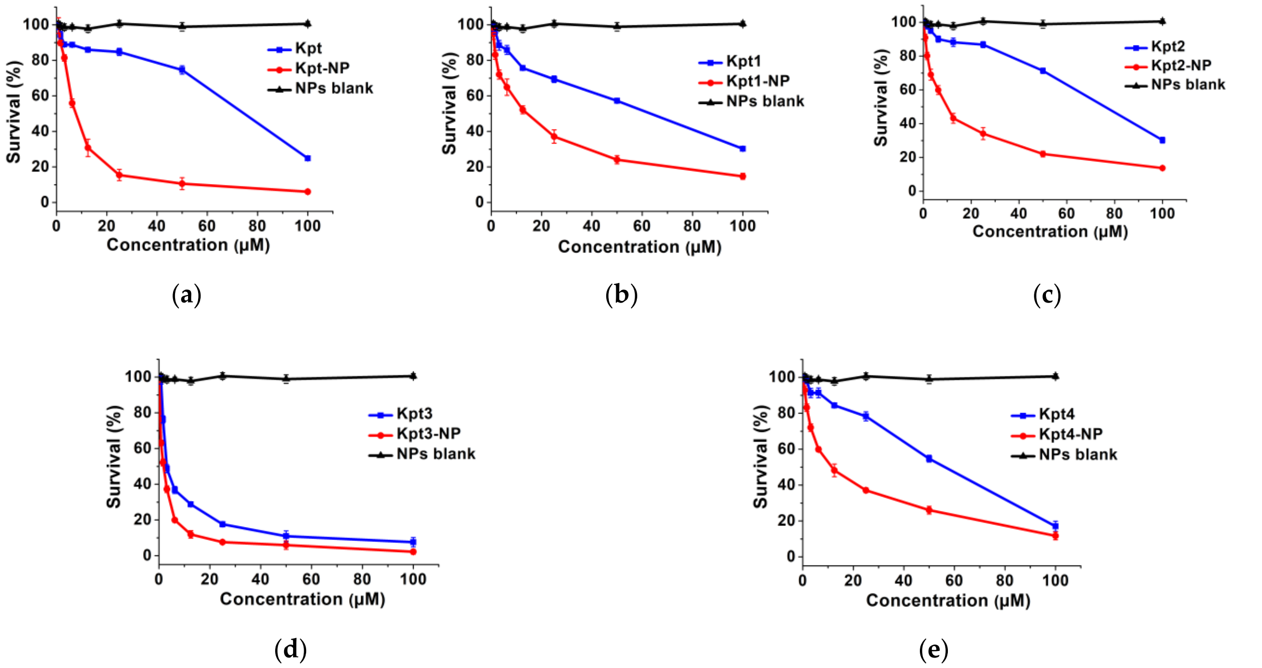

Figure S1. Survival of H69 cells after incubation during 72 h with: (a) Kpt and Kpt-NP; (b) Kpt1 and Kpt1-NP; (c) Kpt2 and Kpt2-NP; (d) Kpt3 and Kpt3-NP; (e) Kpt4 and Kpt4-NP. Each point shows mean ± SD (n=3).

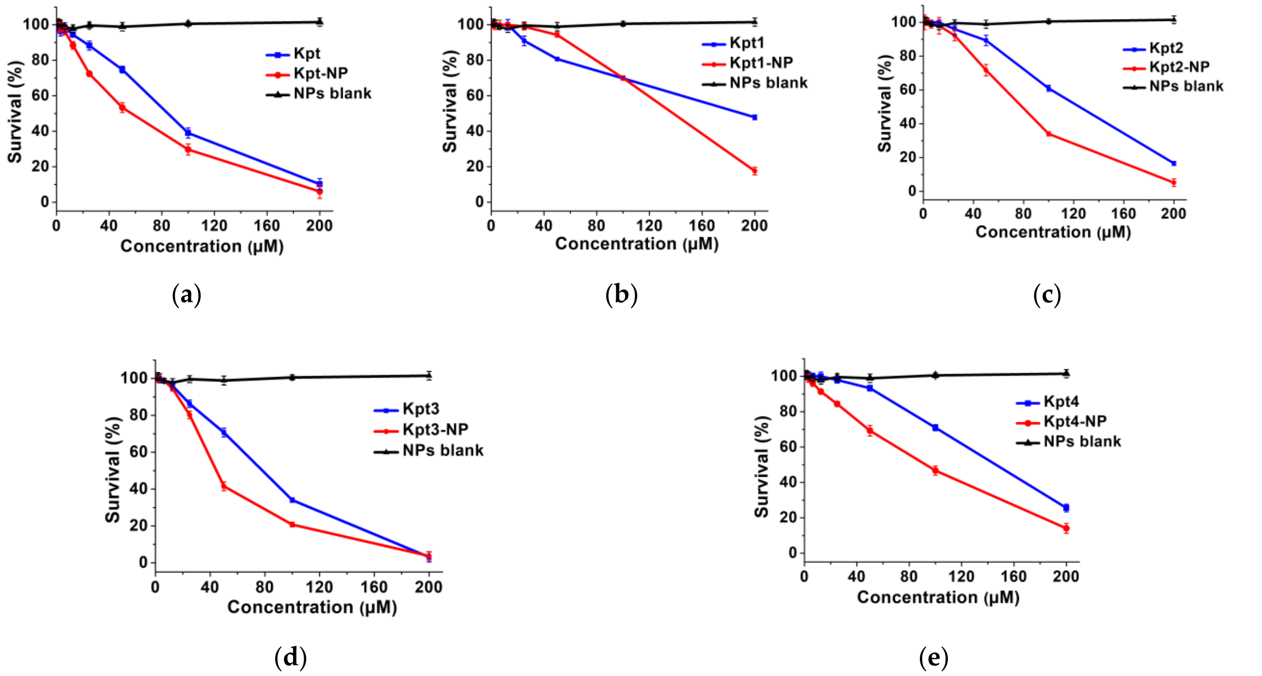

Figure S2. Survival of MCF-7 cells after incubation during 72 h with: (a) Kpt and Kpt-NP; (b) Kpt1 and Kpt1-NP; (c) Kpt2 and Kpt2-NP; (d) Kpt3 and Kpt3-NP; (e) Kpt4 and Kpt4-NP. Each point shows mean ± SD (n=3).
